# Protecting Children From Harmful Food Marketing: Options for Local Government to Make a Difference

**Published:** 2011-08-15

**Authors:** Jennifer L. Harris, Samantha K Graff

**Affiliations:** Rudd Center for Food Policy and Obesity, Yale University; Public Health Law & Policy, Oakland, California

## Abstract

The obesity epidemic cannot be reversed without substantial improvements in the food marketing environment that surrounds children. Food marketing targeted to children almost exclusively promotes calorie-dense, nutrient-poor foods and takes advantage of children's vulnerability to persuasive messages. Increasing scientific evidence reveals potentially profound effects of food marketing on children's lifelong eating behaviors and health. Much of this marketing occurs in nationwide media (eg, television, the Internet), but companies also directly target children in their own communities through the use of billboards and through local environments such as stores, restaurants, and schools. Given the harmful effect of this marketing environment on children's health and the industry's reluctance to make necessary changes to its food marketing practices, government at all levels has an obligation to act. This article focuses on policy options for municipalities that are seeking ways to limit harmful food marketing at the community level.

## Introduction

The prevalence of childhood obesity in the United States imposes a major burden on society in health care costs and children's physical and mental health (1). Meanwhile, the food industry spends massive amounts of money marketing calorie-dense, nutrient-poor foods, and its marketing specifically targets children (2). The obesity crisis cannot be solved without dramatic changes to the obesogenic marketing environment that surrounds children (3).

The White House Task Force on Childhood Obesity has called for immediate action: "Key actors — from food and beverage companies, to restaurants, food retailers, trade associations, the media, government and others — all have an important role to play in creating a food marketing environment that supports, rather than undermines, the efforts of parents and other caregivers to encourage healthy eating among children and prevent obesity" (4). Given government's fundamental obligation to advance public health, lawmakers at all levels must take the lead to change this toxic environment and shield children from exposure to marketing of food products that contribute to the obesity crisis (3). Although the federal government has jurisdiction to regulate national media and the First Amendment to the US Constitution limits what government at any level can do to restrict advertising, municipalities do have constitutionally viable options to protect children from the harmful food marketing that permeates their communities.

In this article, we describe ways in which food companies market calorie-dense, nutrient-poor foods directly to children in national media and local communities. We present evidence that this marketing negatively affects children's diets, increases children's risk for obesity and obesity-related illness, and takes advantage of youths' unique vulnerabilities. We then discuss limitations in food industry self-regulatory initiatives that address these concerns. Next, we present the legal doctrine that balances government's obligation to promote public health against the liberty interests of individual citizens and organizations. Finally, we highlight potentially viable local policy options to restrict the marketing of obesogenic food to children.

## Food Marketing Targeting Children

In 2006, the food industry spent more than $1.6 billion on marketing to youth, including $900 million in marketing aimed directly at children younger than 12 years and designed specifically to increase positive attitudes and preferences for its products (2). Approximately half ($514 million) was spent on television advertising and other forms of national media, including the Internet, print, and radio. Children's exposure to these advertisements is considerable. For example, the average child aged 6 to 11 years views 13 television food advertisements daily (5). Approximately 400,000 children spent more than 60 minutes per month playing games and viewing branded content promoting high-sugar cereals on General Mills's millsberry.com, which was closed down in April 2011 as a result of pressure from public health advocates (6).

Companies also spend considerable sums to reach children directly in their local communities, in stores, restaurants, schools, and almost anywhere children spend their time. These locally based food marketing practices include product packaging, signs, and promotions in stores that appeal specifically to children ($106 million spent in the United States in 2006), marketing to children in schools ($73 million), and local child-focused events ($30 million) (2). Additionally, food companies spend $127 million on premiums, cross-promotion licenses, athletic sponsorships, celebrity fees, and philanthropic tie-ins. These programs increase products' appeal to children by associating foods with popular cartoon characters, sports, and entertainment celebrities and events, and even charities (eg, Girl Scouts [Girl Scouts of the USA, New York, New York]) (7). Similarly, fast-food restaurants spent $360 million in the United States in 2006 on toy giveaways (2). In 2009, the average child viewed 262 television advertisements (5 per week) that encouraged them to visit their local fast-food restaurant for the toy or other promotion in children's meals (8).

Locally based marketing practices are more difficult to measure quantitatively because they vary widely by location; however, reports document the extent of child-targeted marketing in communities and schools. For example, in supermarkets, high-sugar cereals marketed directly to children are more likely to be placed on lower and middle shelves (ie, children's eye level) and be featured in special store displays and price promotions (6). Products featuring youth-oriented cross-promotions on packaging in the supermarket increased by 78% during 2006-2008 (7). In schools, where children provide a captive market for advertisers, examples of food marketing include sponsored incentives (eg, rewarding children with free pizza for reading books); fundraising programs in which schools receive funds when families purchase food products and give proof of purchase to the school; branded food items served in school cafeterias, stores, and vending machines; corporate logos on scoreboards, book covers, and team jerseys; and sponsored curricula with branded content (9). Although less overt than traditional media advertising, school-based marketing practices are designed specifically to increase children's affinity and desire for companies' products by increasing familiarity and positive associations with the brands (10).

Food companies expend these prolific marketing efforts almost exclusively to promote foods that children should consume only occasionally and in limited quantities. On television, 98% of food advertisements watched by children promote products high in fat, sugar, or sodium (11). In all forms of marketing targeted to children, calorie-dense, nutrient-poor foods predominate (3). Breakfast cereals are most frequently marketed directly to children, representing 25% of all child-targeted food marketing in 2006 (2). Moreover, cereal companies choose to market products to children that contain 85% more sugar, 60% more sodium, and 65% less fiber than the products they market to adults (6). Restaurants are the second most frequent food category marketed to children (2). In 2007, fast-food restaurants represented 22% of all television food advertisements viewed by children, an increase of 12% from 2003 (5). Child-targeted spending to market beverages — including carbonated beverages, fruit drinks, and juices — sweets and baked goods, and snack foods totaled $376 million in 2006 (2). In contrast, only 4% of all child-targeted food marketing ($38 million) promoted fruits, vegetables, and dairy products.

## Harmful Effects of Food Marketing

Food companies have traditionally argued that their advertising simply encourages children to prefer one brand over another and thus does not contribute to childhood obesity (3). Most research on the effects of television food advertising to children confirms that it increases children's preferences for advertised brands, choices of specific foods after advertisement exposure, and requests to parents for advertised foods (12). More recent research has demonstrated, however, that food marketing also has potentially profound effects on children's overall diet and health. For example, television food advertising increases consumption of any available snack foods during and immediately after exposure, and exposure to commercial television is associated with increased overall calorie consumption, higher body mass index, and reduced fruit and vegetable consumption 5 years later (10). Research has also demonstrated an association between exposure to soft drink advertising and consumption of all sugar-sweetened beverages (13). Marketing can even affect how much children like the taste of advertised foods: preschoolers indicated that snack foods presented in packages with licensed characters tasted better than the same foods in plain packages (14).

Research on the harmful effects of food marketing on broader health-related outcomes (beyond brand preference and attitudes) is in its early stages; however, potentially far-reaching and dangerous effects have been hypothesized (10). Because of its ubiquity, food marketing likely affects children's normative beliefs about the types of foods that are acceptable to eat regularly without adverse consequences, may affect how much children like the taste of advertised foods, and may automatically prime other unrelated goals and behaviors, including children's motivation to engage in unhealthful behaviors.

## Children's Unique Vulnerability

Child advocates also question the ethics of marketing practices targeted to children who cannot yet defend against their influence (3). Research consistently demonstrates that until the age of 8 years, most children do not possess the necessary cognitive skills to understand that advertising is not just another source of information and presents a biased point of view (12). Although older children and adolescents understand the intent of advertising, they do not regularly act on that knowledge nor do they attempt to counteract its influence. Resisting advertisements for the highly tempting products commonly promoted also requires the ability to weigh long-term health consequences of consumption against short-term rewards, an ability that is not fully developed until the early 20s (15). Finally, marketing practices that persuade indirectly (eg, through logo placements, associations with popular characters and movies, and Internet games) are designed to create lifelong customers by imprinting brand meaning into the minds of young children (10). Before children know better, they have learned to love the products they encounter most frequently and associate with positive experiences.

Evidence also exists that food companies disproportionately target advertising for high-calorie, nutrient-poor foods to black and Hispanic communities, where youth are most vulnerable to obesity and obesity-related disease (16). For example, billboards for fast-food and sugar-sweetened beverages appear substantially more often in low-income black and Hispanic neighborhoods (17), and fast-food outlets in low-income black communities are more likely to promote less healthful menu items (18).

## Food Industry Initiatives to Address These Public Health Concerns

The food industry appears to have heard concerns raised by the public health community about child-targeted marketing practices. In 2006, the Children's Food and Beverage Advertising Initiative (CFBAI), an industry-sponsored program purportedly designed to improve food-marketing practices, was launched (19). Sixteen of the largest food companies that market to children have joined CFBAI and implemented pledges to market only "better-for-you" foods in child-targeted media. However, exclusions and limitations demonstrate that these pledges are unlikely to produce substantial changes to existing marketing practices. For example, participating companies have created their own definitions of "better-for-you" foods that include products dietitians may regard as unhealthful, including high-sugar cereals, juice drinks made of 10% fruit juice with 16 grams of sugar per 6-ounce serving, and even certain flavors of toaster pastries. Similarly, participating companies have declared that widely used forms of marketing designed specifically to appeal to children are not child-targeted advertising and thus not subject to limitations, including product packaging and other types of marketing to children that occur in stores or restaurants, advertising on prime-time television shows popular with children but also viewed by a broader audience, and food and beverage displays in schools.

Recent evaluations of the effects of the CFBAI pledges on the volume and types of foods advertised to children demonstrate limited changes in the foods advertised to children (6,8,20). Public health professionals have suggested that industry self-regulatory efforts (eg, CFBAI) provide more public relations benefit to the food industry than real health benefits for children and that overreliance on such efforts could exacerbate the childhood obesity crisis (21-23).

## Government's Role in Advancing Public Health

Because the food marketing environment contributes to the health crisis facing our nation's children and because members of the food industry appear unlikely to voluntarily make the considerable changes required to improve this environment, government at all levels has an obligation to intervene where it can. In our constitutional democracy, a core responsibility of government is to protect and promote public health — especially among vulnerable populations, including children. Public health is essential to civil society because it provides the general population with basic security and welfare that can be achieved only through collective action (24).

A common refrain among opponents of public health regulations is that government should not impede individual liberty to benefit the general public. The US Constitution addresses this concern: provisions of the Bill of Rights and the Fourteenth Amendment — including those regarding free speech, due process, equal protection, and property ownership — mandate that a balance be struck between the government's obligation to serve the general welfare and the interest of individual citizens and organizations in freedom, fairness, and self-determination (25). Therefore, when the government regulates food marketing to children, it must achieve a balance between public and private interests.

Congress and federal agencies (eg, the Federal Trade Commission, the Federal Communications Commission) have purview over media that cross state lines, including television, radio, the Internet, and other digital media. Consequently, policy efforts to limit food advertising in the national media must be initiated at the federal level. However, states and localities have power to take a regulatory stand against many forms of marketing for obesogenic food to youth that occur in their communities, including marketing in retail establishments, restaurants, and schools. In fact, states and their subdivisions have always borne primary responsibility for public health. In the constitutional compact, each state retains police power — the inherent authority to act in the interest of the public's health, safety, and welfare (24). Most states grant their localities a form of home rule, or the ability to legislate on the basis of the police power (26). Because regulating public health is a fundamental police power function, states and home-rule localities have the presumptive authority to pass public health laws as long as the laws do not overstep constitutional bounds and are not preempted (ie, trumped) by the law of a higher jurisdiction.

Accordingly, public health advocates have identified multiple policy proposals that localities can consider to reduce marketing of unhealthful food to children in stores, restaurants, schools, and elsewhere in the community (Table). A limited number of the policies listed have been tested in court, but in light of case law on analogous policies, these all have a reasonable chance of withstanding constitutional scrutiny. A community interested in pursuing any of these policies should involve government attorneys early in the process to ensure the proposal is legally sound in the jurisdiction.

An important role of local government is to serve as a testing ground for new and promising public health initiatives. One of the special features of our constitutional system is that, to paraphrase Justice Louis Brandeis, our states and localities serve as laboratories of democracy, testing new social and economic experiments that can be studied, adapted, and honed to benefit other jurisdictions. Given the recent attention to food marketing as a significant contributor to childhood obesity, many of the policies listed in the Table are untried or just starting to be tried. Therefore, we do not yet know which will be most effective at limiting marketing of unhealthful foods and ultimately improving children's health. As local governments develop and implement policies to address the marketing of unhealthful foods in their communities, it is critical that they form partnerships to conduct research and generate knowledge about the effectiveness of their policies and to transfer that knowledge to other municipalities (27). Protecting children from the harmful effects of food marketing requires a range of policy interventions at all levels of government — and ultimately a change in social norms of acceptable behavior.

## Conclusion

Action must be taken to change the obesogenic environment that surrounds children, and food marketing is a key contributor to that environment. Initial actions by the food industry do not reflect a genuine commitment to reversing the effects of persistent and prolific marketing programs directly targeted to children, which continually reinforce the rewards of consuming nutrient-poor, calorie-dense foods. Accordingly, both the federal and local governments have an obligation to act. Municipalities can play a critical role in developing, implementing, and evaluating policies to improve the marketing environment for children in their own communities and across the country.

## Acknowledgments

This work was supported by grants from the Robert Wood Johnson Foundation through the National Policy and Legal Analysis Network to Prevent Childhood Obesity, a project of Public Health Law & Policy, and the Rudd Center for Food Policy and Obesity, and by the Rudd Foundation. The authors thank Professor Mark Aaronson for his insights on framing the manuscript. This article highlights ideas generated and conclusions reached at the Symposium on Ethical Issues in Interventions for Childhood Obesity, sponsored by the Robert Wood Johnson Foundation and Data for Solutions, Inc.

## Figures and Tables

**Table.** Local Policy Options to Restrict Marketing of Unhealthful Foods to Children^a^

**Location**	Policy Options
Supermarkets, convenience stores, and other retail outlets	Impose excise taxes or fees on sugar-sweetened beverages, and earmark a portion or all of the revenue to fund obesity prevention programs. Require "healthy checkout aisles," free of obesogenic food and beverages. Prohibit food sales in nonretail food outlets (eg, sporting goods stores, toy stores). Limit sales of obesogenic food and beverages near schools before, during, and immediately after the school day. Regulate the pricing of obesogenic food and beverages (eg, set minimum prices). Limit the total amount of store window space that can be covered by signs. To avoid potential First Amendment violations, the policy should apply to all signs no matter the message and should be based on non–speech-related considerations such as minimizing visual clutter. Require food retailers to obtain a license that comes with conditions limiting in some way the sale of obesogenic food and beverages.
Restaurants and other food service establishments	Set nutrition standards for children's meals that include a toy or other incentive item. Enact a menu labeling law that is identical to the federal law (thus enabling local enforcement) and/or that applies to food service establishments that are not covered under the federal law. Prohibit new fast-food restaurants from opening near schools. Restrict the number or density of fast-food restaurants. Ban drive-through windows. Prohibit use of trans fats in restaurant food. Set procurement standards for government-run food facilities. Implement a healthy restaurant certification program that encourages restaurants to reduce the sale and advertising of obesogenic food and beverages to children.
Schools^b^	Ban the sale of obesogenic food and beverages on school property. Ban all food advertising on school property or ban advertising on school property for foods that are not allowed to be sold on campus. Include provisions in vending contracts limiting the sale and advertising of obesogenic food and beverages on school property. Prohibit fundraisers that entail selling obesogenic food and beverages. Implement closed campus policies to reduce student exposure to obesogenic food marketing.
Elsewhere in the community	Ban all commercial billboards except those located on the site of the advertised establishment. To avoid potential First Amendment violations, the ban should be based on non–speech-related considerations such as traffic safety or esthetics. Include provisions in vending contracts limiting the sale and advertising of obesogenic food and beverages in parks and other public venues that are frequented by children.

a This list expands on a list originally developed by the members of the Food Marketing to Children Workgroup's local subcommittee, including Samantha Graff.

b School districts, rather than local legislatures, usually have the authority to enact policies that restrict marketing of unhealthful foods in public schools.
